# Supplementary material for: Complex strain evolution of polar and magnetic order in multiferroic BiFeO3 thin films
Source: Nat Commun. 2018 Sep 21;9:3764. doi: 10.1038/s41467-018-06190-5 (PMC6155110; doi:10.1038/s41467-018-06190-5)
Supplement: Supplementary file 1 — Supplementary Information [file 41467_2018_6190_MOESM1_ESM.pdf]

Supplementary Information for:

Complex Strain Evolution of Polar and Magnetic Order in Multiferroic $BiFeO_3$ Thin Films

Chen *et al.*

Supplementary Note 1. Structural characterization of $BiFeO_3$ thin films using X-ray diffraction

At room temperature, bulk $BiFeO_3$ possesses a rhombohedral perovskite structure with a pseudocubic lattice parameter of $a = 3.965$ Å, $\alpha = 89.4°$.[1] $SrTiO_3$ has an cubic structure with $a = 3.905$ Å and $GdScO_3$ is orthorhombic with a_0 = 5.488 Å, b_0 = 5.746 Å, and c_0 = 7.934 Å.[2] The orthorhombic unit cell can be described with a pseudocubic space group, in which the $[001]_O$, $[100]_O$, and $[010]_O$ orthorhombic directions correspond to the $[001]$, $[1\bar{1}0]$, and $[110]$ pseudocubic directions, respectively; therefore, $GdScO_3$ $(010)_O$ substrates are akin to a (110)-oriented cubic perovskite substrate and result in epitaxial growth of (110)-oriented $BiFeO_3$ films.[3] A representative $\theta - 2\theta$ XRD pattern of a ~12 nm thick $BiFeO_3$ / $GdScO_3$ $(010)_O$ heterostructure (Supplementary Fig. 1) reveals only *hh*0-diffraction peaks for the film and the substrate suggesting epitaxial growth without impurity phases. The thickness fringes apparent near the $BiFeO_3$ diffraction peaks indicate the high quality of the films. The out-of-plane lattice parameter of the film is measured to be ~2.825 Å, larger than the (110) *d*-spacing of bulk $BiFeO_3$ ($d_{110,bulk}$ = 2.804 Å), suggesting that the film is under average in-plane compressive strain.

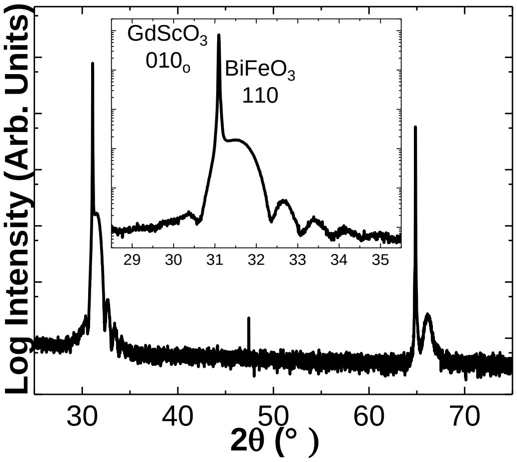

Supplementary Figure 1 | X-ray diffraction characterization. $\theta - 2\theta$ scan of a representative 12 nm $BiFeO_3$ film grown on a $GdScO_3$ $(010)_O$ substrate.

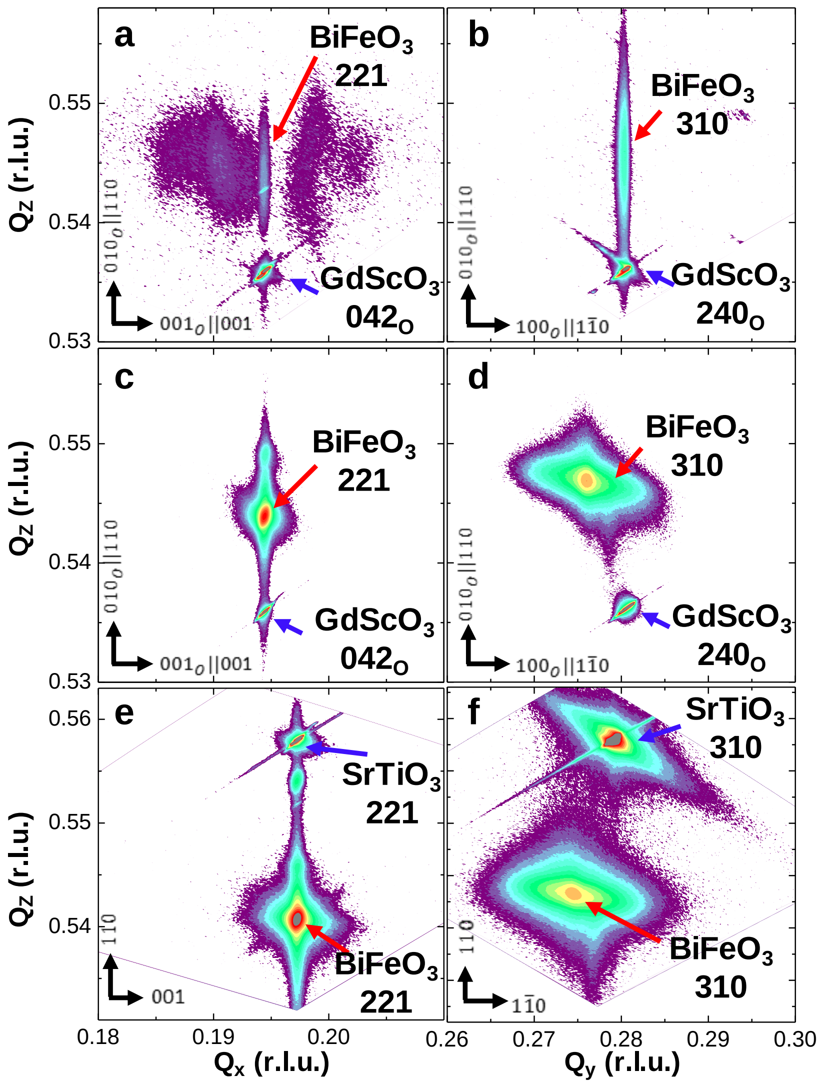

Supplementary Figure 2│ Reciprocal space mapping characterizations. Reciprocal space mappings for a 12 nm $BiFeO_3$/ $GdScO_3$ $(010)_O$ heterostructure about the substrate (film) **a,** 042_O- (221-), **b,** 240_O- (310-) diffraction conditions. RSMs for a 70 nm $BiFeO_3$/$GdScO_3$ $(010)_O$ heterostructure about the substrate (film) **c,** 042_O- (221-), **d,** 240_O- (310-) diffraction conditions. RSMs for a 70 nm $BiFeO_3$/$SrTiO_3$ (110) heterostructure about the substrate **e,** 221-, **f,** 310- diffraction conditions.

To investigate the domain structure and strain state of the various $BiFeO_3$ films, off-axis reciprocal space mapping (RSM) studies were conducted about the 221- (*i.e.*, 042_O) and 310- (*i.e.*, 240_O) diffraction conditions of the $SrTiO_3$ ($GdScO_3$) substrates (Supplementary Fig. 2). For the 12 nm thick film on $GdScO_3$ $(010)_O$, the 221- (Supplementary Fig. 2a) and 310- (Supplementary Fig. 2b) diffraction peaks from the film and substrate possess the same in-plane values, confirming that the thin film is coherently strained to the substrate despite the rather large in-plane structural

anisotropy (Supplementary Fig. 2c). (Note that satellite peaks due to 180° stripe domains are observed in the 042_O-diffraction conditions.)[3] For the 70 nm thick films on $GdScO_3$ $(010)_O$ (Supplementary Figs. 2c-d) and $SrTiO_3$ (110) (Supplementary Figs. 2e-f), only a single $BiFeO_3$ diffraction peak is observed in the corresponding 221- and 310-diffraction conditions, indicating that the films exhibit only a single ferroelastic domain. In addition, the position of the 221-diffraction condition of the $BiFeO_3$ along the [001] has an identical in-plane value with that of the corresponding in-plane diffraction condition of the $GdScO_3$ and $SrTiO_3$ substrate, while the 310-diffraction condition of the $BiFeO_3$ peak has a smaller Q_x value than that of the corresponding diffraction condition of the substrate along $[1\bar{1}0]$. That is, the $BiFeO_3$ films are uniaxially strained (i.e., coherently strained along the $[001]_O$ (i.e., [001]), but relaxed (or partially strained) along the $[100]_O$ (i.e., $[1\bar{1}0]$) as is typically observed in (110)-oriented perovskite film.

Supplementary Note 2. Piezoelectric force microscopy studies of the (110) films

The single ferroelastic domain state of the $BiFeO_3$ films is further confirmed by piezoelectric force microscopy (PFM). Both in-plane (Supplementary Fig. 3a) and out-of-plane (Supplementary Fig. 3b) PFM images of a 70 nm $BiFeO_3$ film, here shown for growth on a $SrTiO_3$ (110) substrate, exhibit no contrast; consistent with that expected for a monodomain structure.

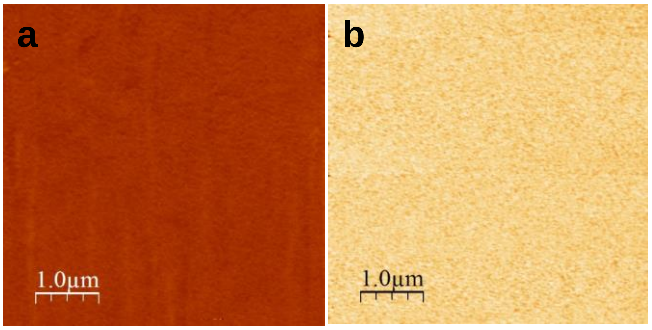

Supplementary Figure 3│ Piezoelectric force microscopy studies. **a,** In-plane and **b,** Out of-plane piezoelectric force microscopy images taken with the cantilever pointed along the $[1\bar{1}0]$ axis of the 70 nm $BiFeO_3$ films on $SrTiO_3$ (110).

Supplementary Note 3. Temperature-dependent X-ray linear dichroism studies

Soft X-ray absorption spectroscopy (XAS)-based linear dichroism studies have been established as an excellent tool to investigate element-specific magnetic properties (*e.g.*, spin orientation, Néel temperature, and magnetic domain structure) of antiferromagnetic materials. XLD is the difference of the absorption of linearly-polarized light, measured at a particular photon energy (in this case corresponding to the Fe $L_{2,3}$ absorption edges), with two orthogonal polarizations. XLD can typically arise from two different origins: magnetic linear dichroism and crystal-field linear dichroism. In the case of multiferroic $BiFeO_3$, the dichroism could arise from both the antiferromagnetism and crystal-field effects due to spontaneous polarization and strain-induced lattice distortion.[4,5] In rhombohedral $BiFeO_3$, which has d^5 high spin ground state of Fe^{3+}, the magnetic linear dichroism mainly shows as variations in peak intensity,[6,7] and the crystal-field effects raise to shifts in peak position.[7,8] A negligible peak shift (< 0.1 eV) between the Fe L_3 spectra with two different polarization of the $BiFeO_3$ heterostructures studied herein indicates a small contribution from the crystal-field mechanism (data for normal incidence, Fig. 1d and for grazing incidence, Supplementary Fig. 4a). The magnetic contribution can be further isolated by temperature-dependent XLD studies since the magnetic linear dichroism is expected to decrease

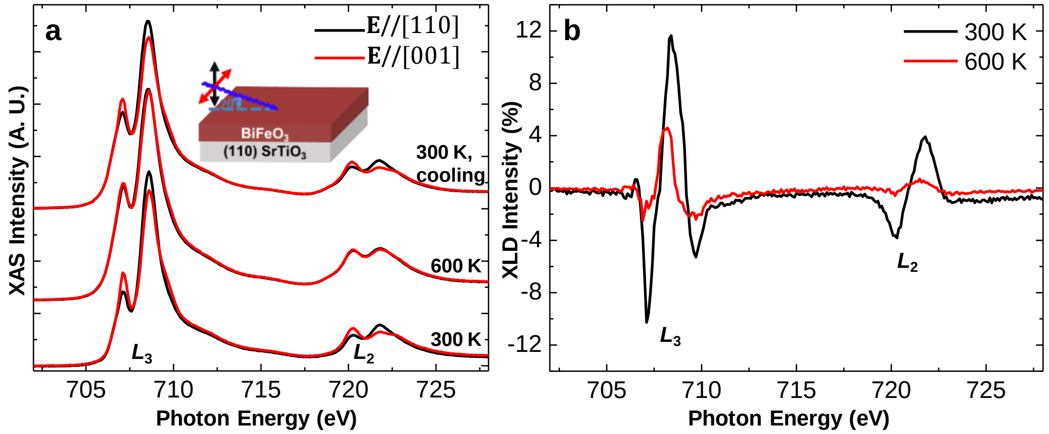

Supplementary Figure 4│ X-ray linear dichroism studies. **a,** X-ray absorption spectroscopy and **b,** X-ray linear dichroism at the Fe $L_{2,3}$ edges at 300 K and 600 Kfor the $BiFeO_3/SrTiO_3$ (110) heterostructures.

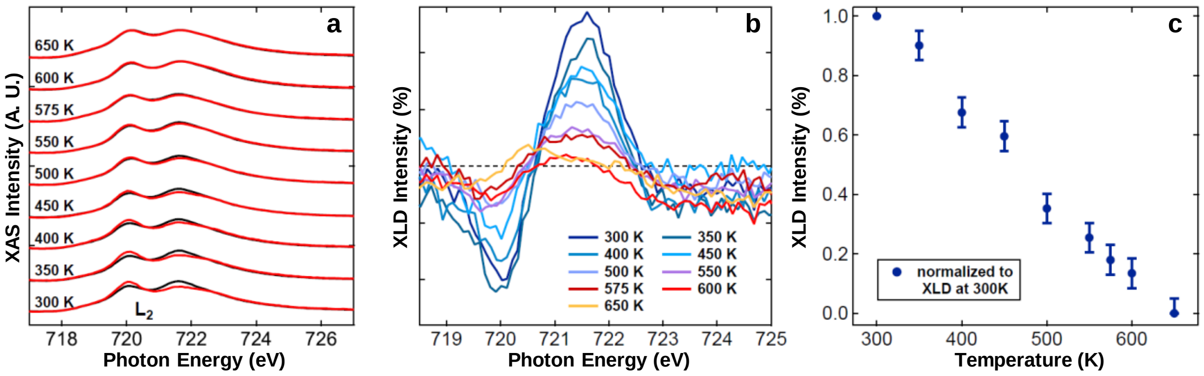

Supplementary Figure 5│ Temperature dependent X-ray linear dichroism studies. Temperature dependent **a,** X-ray absorption spectroscopy and **b,** X-ray linear dichroism at the Fe L_2 edges at different for a $BiFeO_3/SrTiO_3$ (110) heterostructure. The XLD ratio **c,** is normalized to the signal measured at 300K.

when the sample is heated to near the Néel temperature and the magnitude of the magnetic dichroism scales with the average of the square of the magnetic order parameter. In $BiFeO_3$, the crystal-field contribution should persist to well above the Néel temperature since its ferroelectric Curie temperature is well above its Néel temperature. Temperature-dependent XLD at the Fe $L_{2,3}$ edges for the $BiFeO_3$ films found that XLD near T_N is much smaller than that at 300 K (Supplementary Fig. 4b), especially for XLD at the L_2 edge which essentially vanishes at 650 K (Supplementary Fig. 5), indicating that the XLD in our $BiFeO_3$ heterostructures is largely dominated by magnetic origin.[7,9] Reproducibility is demonstrated by the measurements at room temperature obtained before and after, respectively, heating up to 650 K, indicating that there is no film decomposition during heating.

Supplementary Note 4. Atomic multiplet calculations

We have measured the polarization dependent Fe-L_2 XAS spectra of the (110)-oriented $BiFeO_3$ films at room temperature with the Poynting vector of the light being parallel to the $[1\bar{1}0]$, $[001]$, and $[110]$ (Supplementary Fig. 6 and Fig. 7). In this figure, we provide a comparison between the experimental (top) and the simulated (bottom) XAS spectra of the $BiFeO_3$

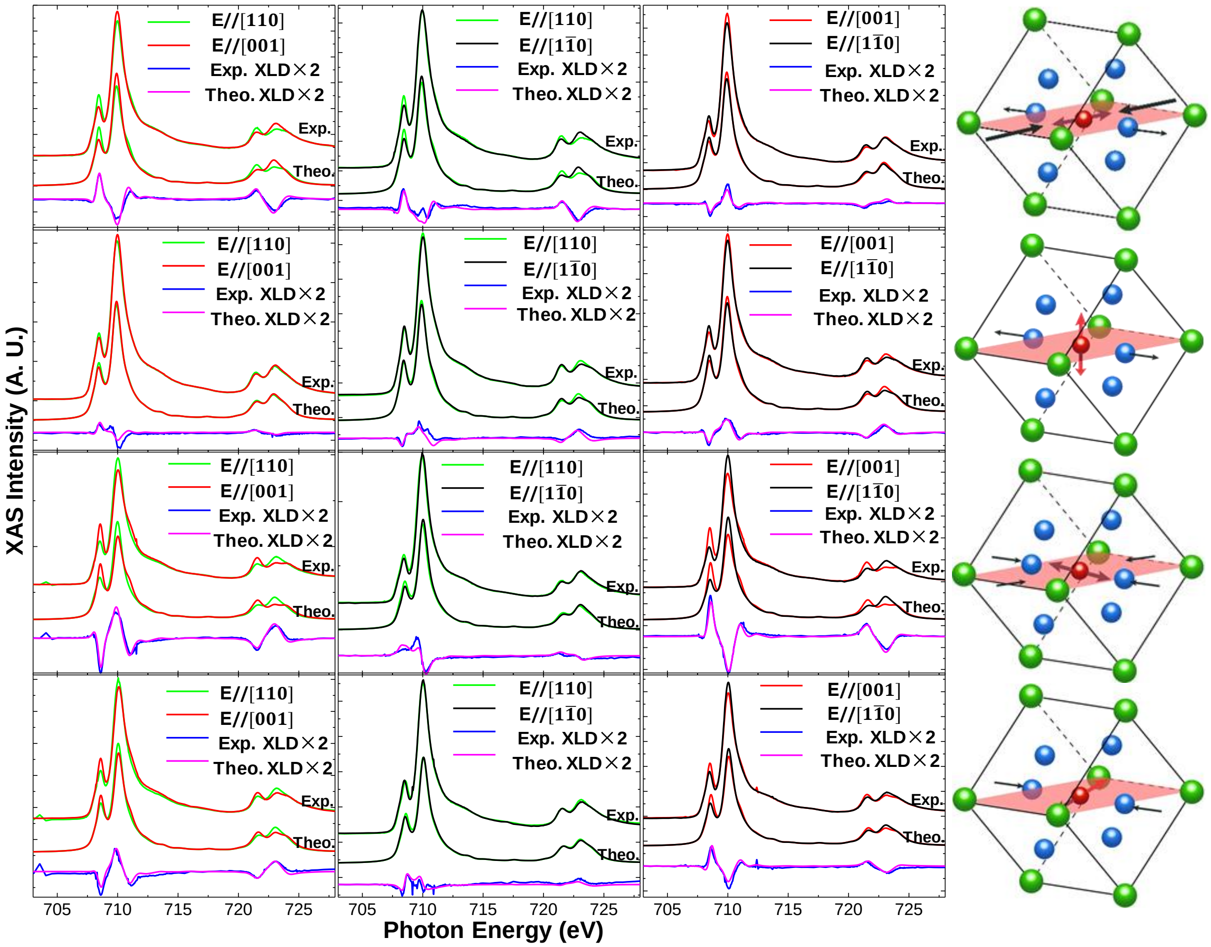

Supplementary Figure 6 │ Linear polarized X-ray absorption spectroscopy studies. Experimental and calculated polarization-dependent Fe $L_{2,3}$ X-ray absorption spectroscopy and X-ray linear dichroism for a **a-c**, 12 nm $BiFeO_3/GdScO_3$ $(010)_O$, **d-f**, 70 nm $BiFeO_3/GdScO_3$ $(010)_O$, **g-i**, 12 nm $BiFeO_3/SrTiO_3$ (110), and **j-l**, 70 nm $BiFeO_3/SrTiO_3$ (110) heterostructures with the incident beam parallel to [1$\bar{1}$0], [001], and[110].

thin films (In calculations the crystal filed parameters of D_{2h} symmetry for FeO_6 cluster are: 0 meV, -50 meV of Δe_g, and 0 meV, -10 meV of Δt_{2g} for $BiFeO_3$ films on $GdScO_3$ and $SrTiO_3$, respectively; 20 meV, -90 meV, 50 meV of D_u for the $BiFeO_3$ films on $GdScO_3$, the $BiFeO_3$ thinner film and thinker film on $SrTiO_3$, respectively; 50 meV, 100 meV of $E_{x^2y^2/z^2}$mix for $BiFeO_3$ films on $GdScO_3$ and $SrTiO_3$, respectively). One can see that the calculations reproduce the main features at both the $L_{2,3}$ edges of the experimental spectral for the $BiFeO_3$ films under various strain and AFM states with **L** parallel to [1$\bar{1}$0], [110], and [001] for the 12 nm $BiFeO_3$ films on $GdScO_3$ $(010)_O$ (Supplementary Figs. 6, 7a-c), 70 nm $BiFeO_3$ films on $GdScO_3$ $(010)_O$ (Supplementary

Figs. 6, 7d-f), and 12 nm $BiFeO_3$ films on $SrTiO_3$ (110) (Supplementary Figs. 6, 7g-i), respectively. For 70 nm $BiFeO_3$ films on $SrTiO_3$ (110) (Supplementary Figs. 6, 7j-l), the spectra can be nicely reproduced by with **L** deviated from in-plane [001] by 35° towards the out-of-plane direction (i.e., the $[11\bar{2}]$ and $[112]$).

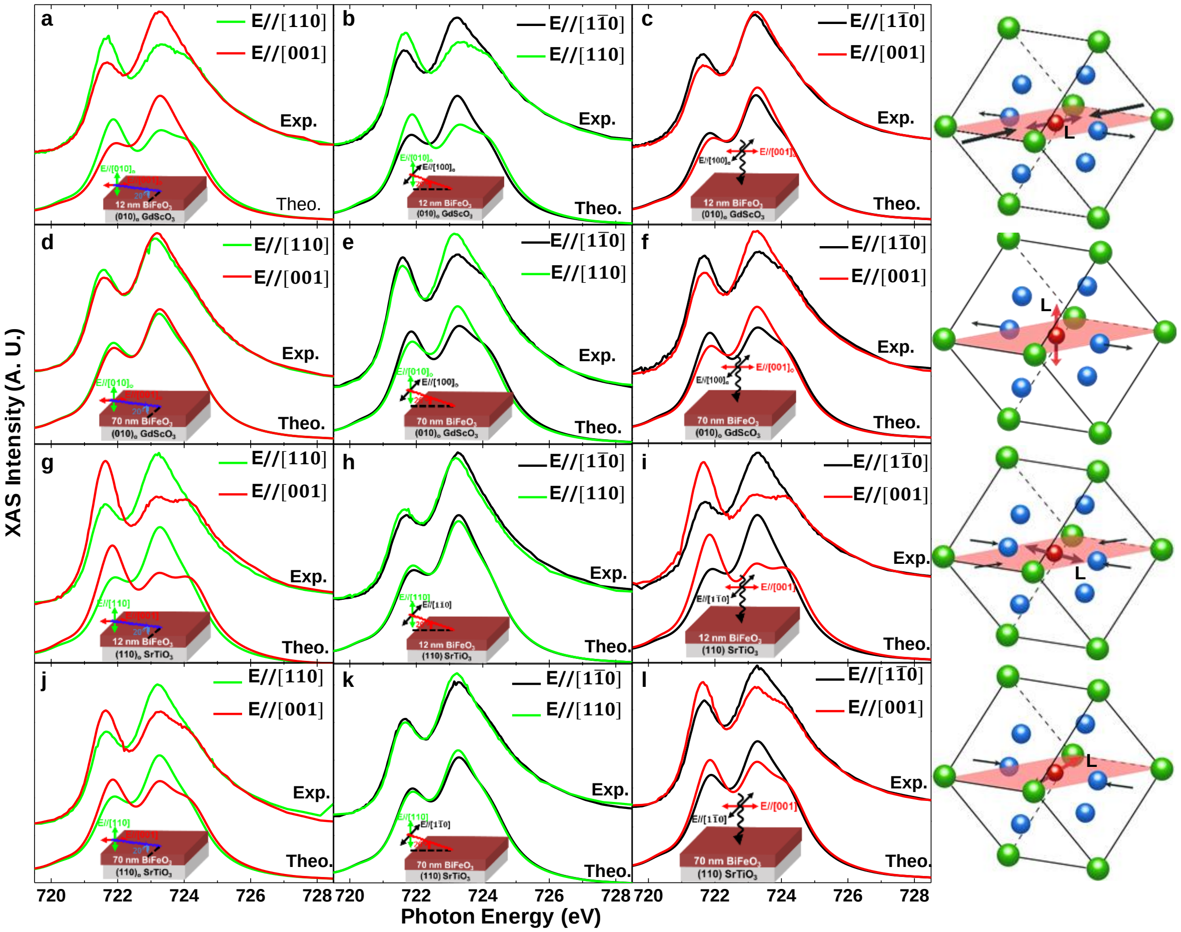

Supplementary Figure 7 | Linear polarized X-ray absorption spectroscopy studies. Experimental and calculated polarization-dependent Fe L_2 XAS spectra for a **a-c**, 12 nm $BiFeO_3/GdScO_3$ $(010)_O$, **d-f**, 70 nm $BiFeO_3/GdScO_3$ $(010)_O$, **g-i**, 12 nm $BiFeO_3/SrTiO_3$ (110), and **j-l**, 70 nm $BiFeO_3/SrTiO_3$ (110) heterostructures with the incident beam parallel to $[1\bar{1}0]$, [001], and[110].

Supplementary Note 5. Scanning transmission electron microscopy studies

High-angle annular dark-field (HAADF) and bright field (BF) scanning transmission electron microscopy (STEM) studies were carried out to characterize the interface abruptness and

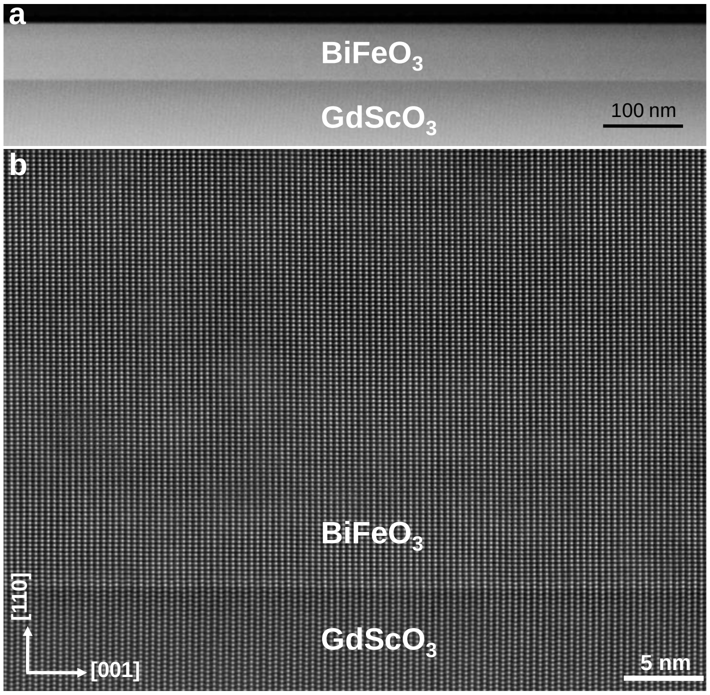

Supplementary Figure 8 │ Scanning transmission electron microscopy studies. a, Low- and **b**, high-resolution high-angle annular dark-field - scanning transmission electron microscopy image of a 70 nm $BiFeO_3/GdScO_3$ $(010)_O$ heterostructure.

polarization of the films. HAADF-STEM images of a representative 70 nm $BiFeO_3/GdScO_3$ $(010)_O$ heterostructure reveals that the heterointerface is coherent along [001] (Supplementary Fig. 8). The Bright Field (BF-STEM) image (Supplementary Figs. 9a,b) show the Fe-cation and O-anion displacements. With all the atoms, including oxygen, imaged in the BF-STEM image, the polar direction of the (110)-oriented $BiFeO_3$ film can be determined straightforwardly. A schematic illustration of the atomic positions (Supplementary Fig. 9b, where the green circles denote Bi, red circles denote Fe, and blue circles denote O) is provided. The atomic structure of the $BiFeO_3$ crystal, as viewed along the [110] zone axis, is shown for comparison (Supplementary Fig. 9c). One can see that the atom configurations in the real film (Supplementary Fig. 9b) are essentially the same as the idealized positions in the schematic (Supplementary Fig. 9c). In turn, this directly confirms that the (110)-oriented films exhibit a similar polarization direction to the

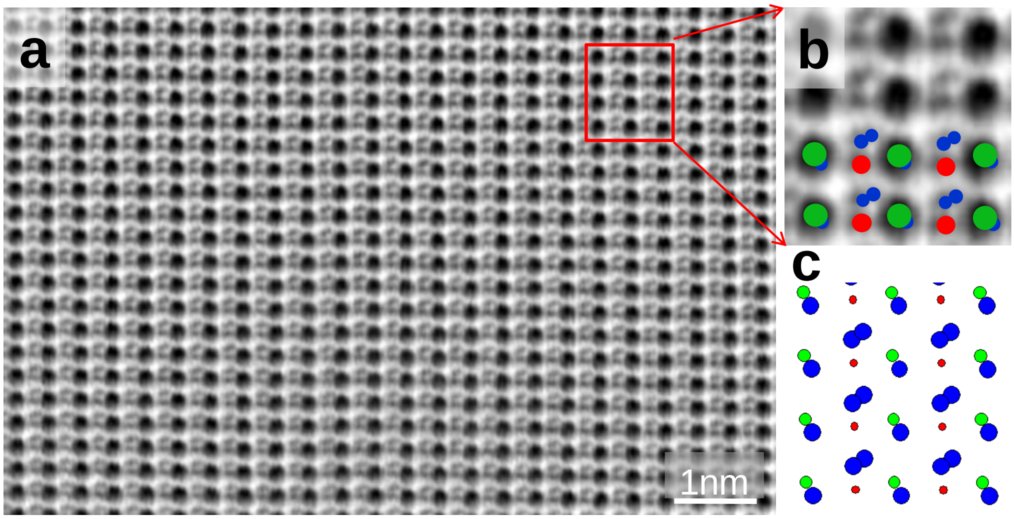

Supplementary Figure 9 | Scanning transmission electron microscopy studies. a, Bright field - scanning transmission electron microscopy image of the same $BiFeO_3$ film in Supplementary Fig. 6. **b**, Magnified image of the area within the red box to show the Fe-cation and O-anion displacement details. **c**, The atom structure of the $BiFeO_3$ crystal viewed along the [110] projection.

bulk and that it has not rotated dramatically in these films. Similar polarization mapping studies have been carried out on films on $SrTiO_3$ (110), and a similar polarization direction is found.

Supplementary Note 6. Polarization-electric field loop measurements

Polarization-voltage hysteresis loop measurements (Supplementary Fig. 10) were performed on the (110)-oriented films grown on $SrRuO_3$-buffered $GdScO_3$ $(010)_O$ substrates to further confirm the polarization orientation determined from above STEM polarization mapping. The hysteresis loops were measured by using a Precision Multiferroic Tester (Radiant

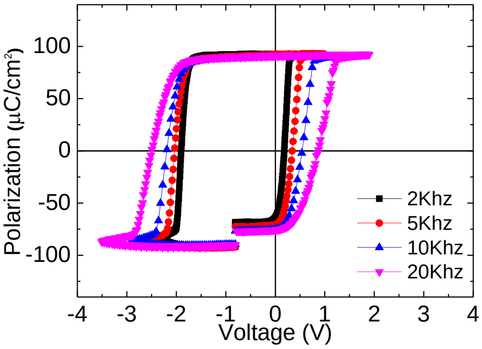

Supplementary Figure 10 | Polarization-voltage hysteresis loop measurements. Ferroelectric hysteresis loop for a 70 nm $BiFeO_3$/ $SrRuO_3$/$GdScO_3$ $(010)_O$ heterostructure.

Technologies). The macroscopic hysteresis loop measurements reveal that the out-of-plane spontaneous polarization of the films grown is ~ 90 $\mu C/cm^2$, close to the value in (110)-oriented films grown on $SrRuO_3/SrTiO_3$ in previous studies.[10] Again, confirming that the film's polarization is essentially pointing along the [111] and has not rotated dramatically.

Supplementary Note 7. *Ab initio* calculations

The *ab initio* calculations adopted a supercell with 2×2×2 cubic perovskite unit cells, containing 40 atoms in total. The three axes of the cell are set to be $[1\bar{1}0]$, [001], and [110], respectively, so as to simulate film grown on the (110) substrates. The atomic structure of the supercell is provided here (Supplementary Figure 11). To reproduce the strain effect, the in-plain [001] and $[1\bar{1}0]$ axes of supercell are fixed while the out-of-plain [110] axis and the atomic positions are fully relaxed.

The evolution of several physical properties of $BiFeO_3$ as a function of misfit strain were then probed (Supplementary Fig. 12). It can be seen that the supercell volume and FeO_6 octahedral volume increase when the in-plane misfit is changed from compressive to zero, then to tensile strain, while the out-of-plane [110] axis length decreases during this process. The decreasing out-of-plane axis is not able to cancel out the increasing in-plane axis, leading to the volume trend.

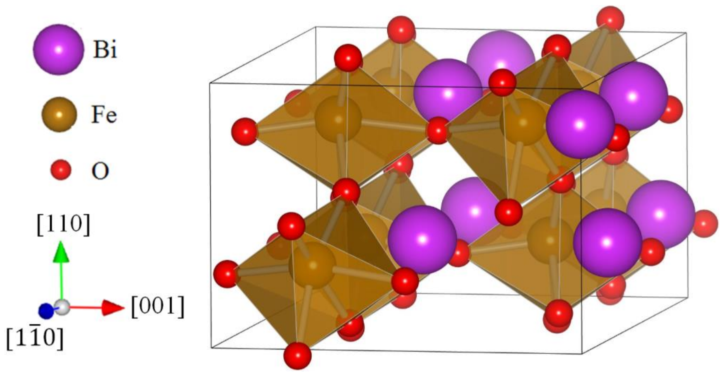

Supplementary Figure 11 | Ab initio calculations. $BiFeO_3$ supercell with 40 atoms. The $[1\bar{1}0]$ and [001] axes are in-plane and the [110] axis is out-of-the-plane. The FeO_6 octahedra are indicated in the space filling view.

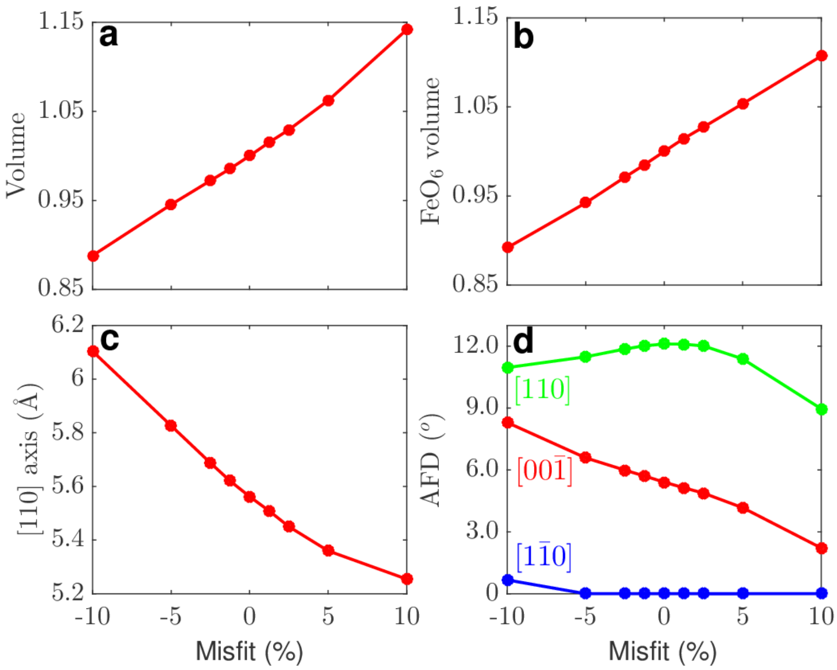

Supplementary Figure 12 | Ab initio calculations. Physical properties as a function of strain for a (110)-oriented $BiFeO_3$ film. **a,** and **b,** are the relative supercell volume and FeO_6 octahedral volume under different strain, compared to the value without strain. **c**, Displays the length of out-of-plane [110] axis. **d**, Displays the antiferrodistortive (AFD) vectors.

These axes lengths are indicators of the Fe-O bonding strength and length. We also find that the rate of change of the FeO_6 octahedral volume is slower than that of the total volume. The nature of the antiferro-distortion (AFD) has a major change in the in-plane [001] component while the other two components change slightly. From zero strain to large tensile strain, the change of the AFD vectors is continuous. There is no phase transition under tensile strain and this is consistent with previous studies.[11,12] From zero strain to large compressive strain, we observe that there is a jump from -5% to -10% in the $[1\bar{1}0]$ component. This indicates there might be a phase transition during this process. This said, we found that the structure maintains a *Cc* symmetry until ~-5% misfit compressive strain, but loses all symmetry by -10% misfit. Despite this, the overall atomic structure shows no remarkable difference as this strain value is increased and, in turn, this means that this symmetry change (loss) does not affect the trend of the electronic polarization **P** and the antiferromagnetic axis **L**, since their directions change very slightly from -5% to -10% misfit.

The displacement of the positive charge center (Fe; Bi) and negative charger center (O) as a function of the misfit strain was also calculated (Supplementary Fig. 13). It can be seen that the in-plane [1$\bar{1}$0] component and the out-of-plane [110] component only have slight changes with strain. The in-plane [001] component increases when strain is changed from -10% (compressive) to 10% (tensile). This indicates the displacement vector rotates at the (1$\bar{1}$0) plane, similar to the **P** vector illustrated in the main text (Fig 3b.)

The evolution of the three Dzyaloshinskii-Moriya interaction (DMI) vectors ($\mathbf{D}_1$, $\mathbf{D}_2$, and $\mathbf{D}_3$) as a function of the misfit strain is also calculated (Supplementary Fig. 14). Their average value ($\mathbf{D}_1$+$\mathbf{D}_2$+$\mathbf{D}_3$)/3 have been provided in the main text (Figure 4a). We see that the [1$\bar{1}$0] component is very minor, consistent with the corresponding AFD component. At large tensile strain, the **D** values are significantly smaller than at zero strain. At large compressive strain, the DMI interaction is primarily determined by the [001] component of the $\mathbf{D}_2$ vector. These values match well with the discussions in the manuscript.

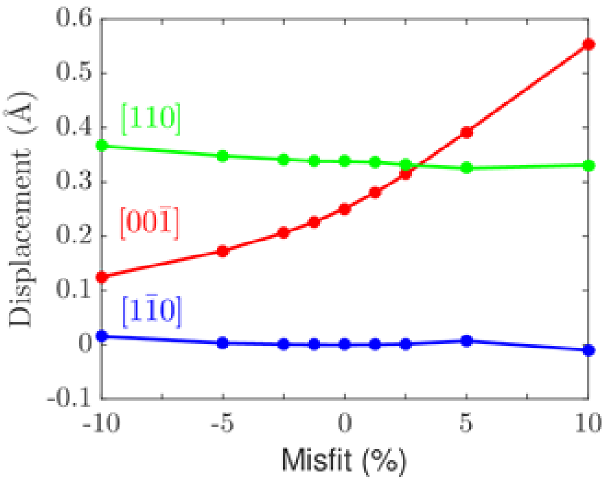

Supplementary Figure 13 | Ab initio calculations. Physical properties as a function of strain for a (110)-oriented $BiFeO_3$ film. **a,** and **b,** are the relative supercell volume and FeO_6 octahedral volume under different strain, compared to the value without strain. **c**, Displays the length of out-of-plane [110] axis. **d**, Displays the antiferrodistortive (AFD) vectors.

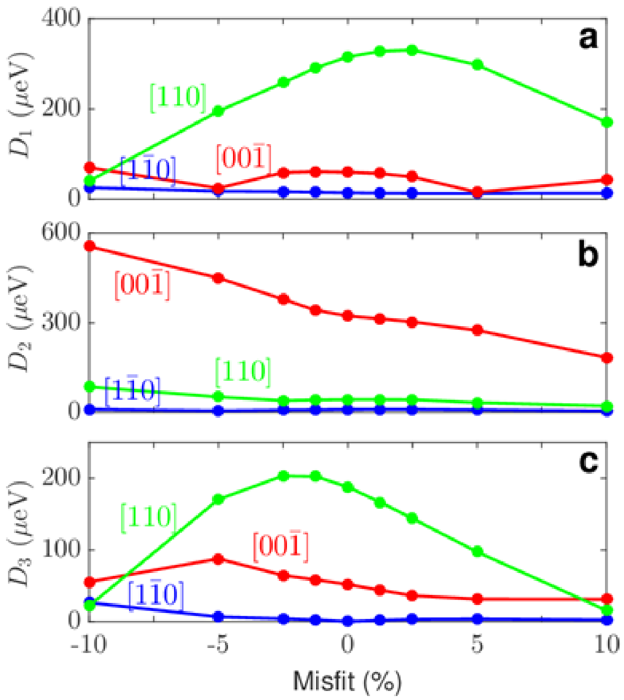

Supplementary Figure 14 | Ab initio calculations. Three DMI vectors ($\mathbf{D}_1$, $\mathbf{D}_2$, and $\mathbf{D}_3$) between the nearest neighboring Fe ions along the [1$\bar{1}$0], [001], and [110] axes, respectively. Each DMI vector have three components in the [1$\bar{1}$0], [001], and [110].

Supplementary Note 8. Magnetic properties measurements

The magnetic hysteresis loops of $Co_{0.9}Fe_{0.1}$ films were measured by longitudinal magneto-optic Kerr effect (MOKE), in which the magnetic field was applied in the optical plane.[13] In addition to the conventional hysteresis loop measurement, we also performed rotation MOKE (ROTMOKE) measurements to determine the magnetic anisotropy of the $Co_{0.9}Fe_{0.1}$ films.[14] For the ROTMOKE measurement, a rotational magnetic field of 700 Oe was applied in the film plane to rotate the Co magnetization. The value of magnetic anisotropy can be retrieved by analyzing the angular difference between the magnetization and the magnetic field. All MOKE and ROTMOKE measurements were carried out at room temperature.

The magnetic hysteresis loops for $Co_{0.9}Fe_{0.1}/BiFeO_3$ heterostructures where magnetic growth field was applied along the [1$\bar{1}$0] are provided here (Supplementary Fig. 15). The

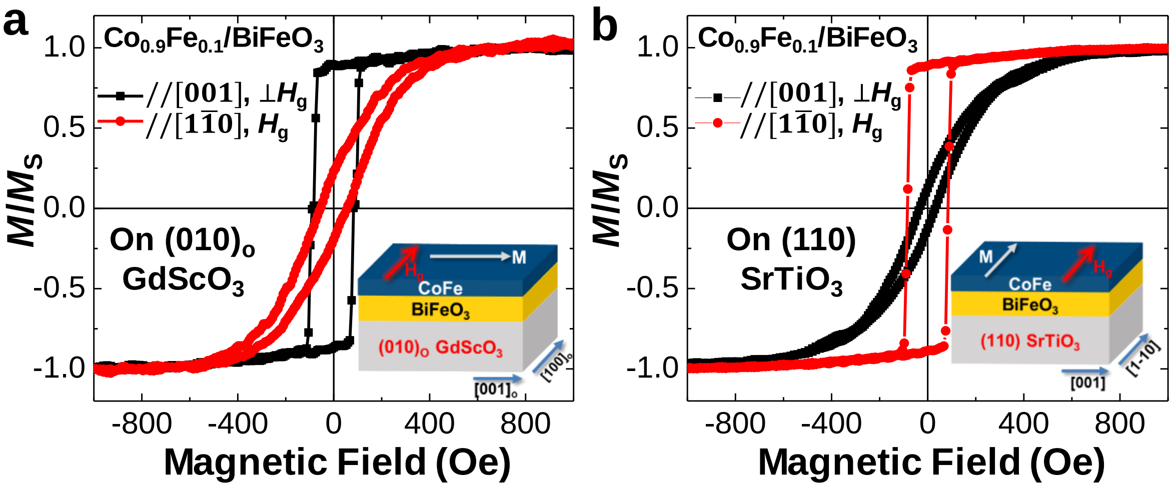

Supplementary Figure 15 | Magnetic properties measurements. Room temperature magnetic hysteresis loops measured in-the-plane of the film for the **a,** Pt/$Co_{0.9}Fe_{0.1}$/$BiFeO_3$/$GdScO_3$ $(010)_O$ and **b,** Pt/$Co_{0.9}Fe_{0.1}$/$BiFeO_3$/(110)$SrTiO_3$heterostructures grown under an applied field of H_G = 200 Oe applied along the in-plane $[1\bar{1}0]$.

ferromagnetic easy axis is along [001] and $[1\bar{1}0]$ for the heterostructures grown on $GdScO_3$ $(010)_O$ (Supplementary Fig. 15a) and $SrTiO_3$ (110) (Supplementary Fig. 15b), respectively. Note that the easy axis direction of the $Co_{0.9}Fe_{0.1}$ layer here does not depend on the growth-field direction. The magnetic hysteresis loops for $Co_{0.9}Fe_{0.1}$ films grown directly on bare (110)-oriented substrates under an applied field of H_g = 200 Oe applied either along the in-plane [001] or $[1\bar{1}0]$ are also provided for comparison (Supplementary Fig. 16). Irrespective of the orientation of the growth field and underlying substrate, the ferromagnetic easy axis is always along [001] for the heterostructures without $BiFeO_3$ layer grown on bare $GdScO_3$ $(010)_O$ and $SrTiO_3$ (110).

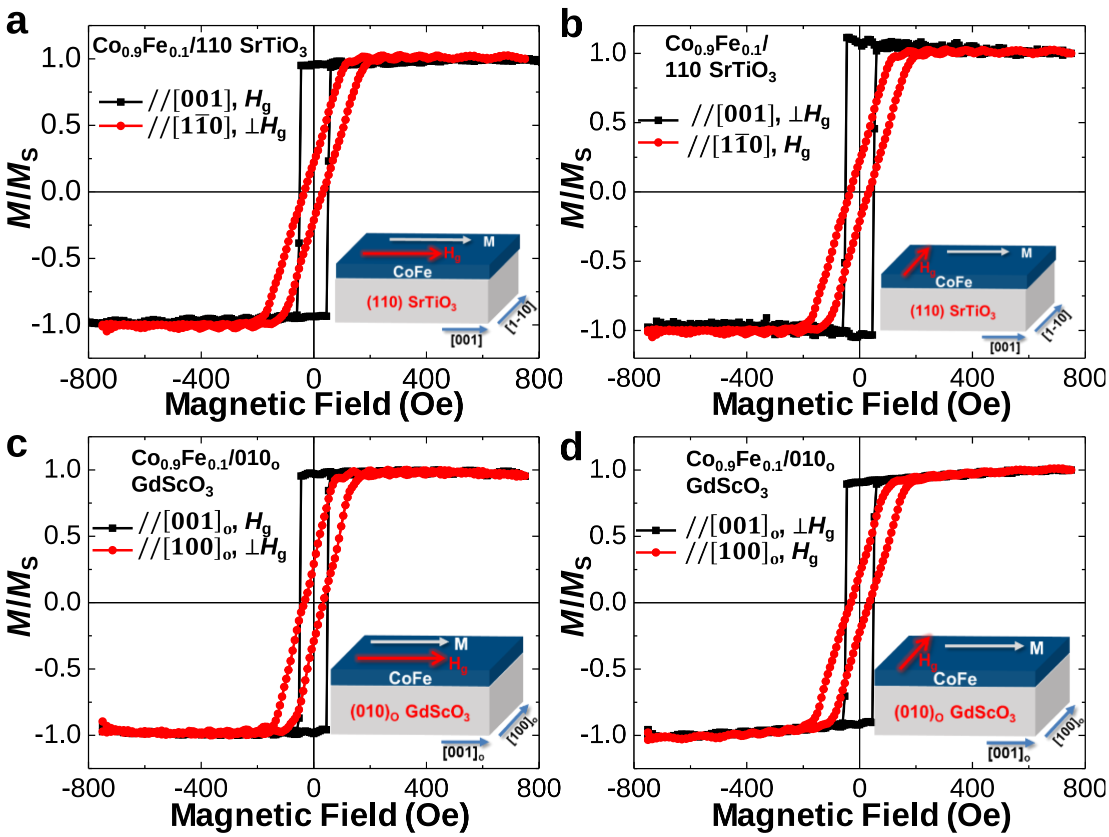

Supplementary Figure 16 | Magnetic properties measurements. Room temperature in-plane magnetic hysteresis loops for the **a,b,** Pt/$Co_{0.9}Fe_{0.1}$/(110)$SrTiO_3$ and **c,d,** Pt/$Co_{0.9}Fe_{0.1}$/$GdScO_3$ $(010)_O$ heterostructures grown under an applied field of H_G = 200 Oe applied along the in-plane **a,c,** [001] and **b,d,** [1$\bar{1}$0].

Supplementary References

1 Kubel, F. & Schimid, H. Structure of a ferroelectric and ferroelastic monodomain crystal of the perovskite $BiFeO_3$. *Acta Crystallogr. B* **46**, 698–702 (1990).

2 Schubert, J. *et al.* Structural and optical properties of epitaxial $BaTiO_3$ thin films grown on $GdScO_3$ (110). *Appl. Phys. Lett.* **82**, 3460-3462 (2003).

3 Chen, Z. *et al.* 180° Ferroelectric Stripe Nanodomains in $BiFeO_3$ Thin Films. *Nano Letters* **15**, 6506-6513 (2015).

4 Zhao, T. *et al.* Electrical control of antiferromagnetic domains in multiferroic $BiFeO_3$ films at room temperature. *Nat. Mater.* **5**, 823-829 (2006).

5 Holcomb, M. B. *et al.* Probing the evolution of antiferromagnetism in multiferroics. *Phys. Rev. B* **81**, 134406 (2010).

6 Kuiper, P. *et al.* X-ray magnetic dichroism of antiferromagnet Fe_2O_3: The orientation of magnetic moments observed by Fe 2*p* X-ray absorption spectroscopy. *Phys. Rev. Lett.* 70, 1549 (1993).

7 Yang, J.-C. *et al.* Electrically enhanced magnetization in highly strained $BiFeO_3$ films. *NPG Asia Mater* **8**, e269 (2016).

8 Kuo, C. Y. *et al*. Single-domain multiferroic $BiFeO_3$ films. *Nat. Commun.* **7**, 12712 (2016).
9 Ko, K.-T. et al. Concurrent transition of ferroelectric and magnetic ordering near room temperature. *Nature Commun.* **2**, 567 (2011).
10 Baek, S.-H. *et al.* The Nature of Polarization Fatigue in $BiFeO_3$. *Adv. Mater.* **23**, 1621-1625 (2011).
11 Prosandeev, S., Kornev, I. A. & Bellaiche, L. Phase Transitions in Epitaxial (-110) $BiFeO_3$ Films from First Principles. *Phys. Rev. Lett.* **107**, 117602 (2011).
12 Dup *et al.* $BiFeO_3$ Films under Tensile Epitaxial Strain from First Principles. *Phys. Rev. Lett.* **106**, 237601 (2011).
13 Li, Q. *et al.* Electrical switching of the magnetic vortex circulation in artificial multiferroic structure of Co/Cu/PMN-PT(011). *Appl. Phys. Lett.* **110**, 262405 (2017).
14 Li, J. *et al.* Design of a vector magnet for the measurements of anisotropic magnetoresistance and rotational magneto-optic Kerr effect. *Rev. Sci. Instrum.* **83**, 033906, (2012).
